# Reverse pupillary block after implantation of a scleral-sutured posterior chamber intraocular lens: a retrospective, open study

**DOI:** 10.1186/s12886-017-0427-1

**Published:** 2017-03-29

**Authors:** Seung Pil Bang, Choun-Ki Joo, Jong Hwa Jun

**Affiliations:** 1 Department of Ophthalmology, Dongsan Medical Center, Keimyung University School of Medicine, #56, Dalseong-ro, Jung-gu, 41931 Daegu South Korea; 2 Department of Ophthalmology and Visual Science, Seoul St. Mary’s Hospital, College of Medicine, The Catholic University of Korea, Seoul, South Korea

**Keywords:** Laser peripheral iridotomy, Reverse pupillary block, Scleral fixed, Vitrectomy

## Abstract

**Background:**

To report the clinical features of patients with reverse pupillary block (RPB) after scleral-sutured posterior chamber intraocular lens (PC IOL) implantation and biometric changes after laser peripheral iridotomy (LPI).

**Methods:**

Eight patients attending our hospital’s ophthalmology outpatient clinic, who developed RPB after implantation of a scleral-sutured PC IOL due to subluxation of the crystalline lens or IOL, were investigated in this retrospective, observational study.

**Results:**

Preoperative evaluations showed angle pigmentation in all cases and iridodonesis in 2 cases. Two subjects had used an α_1A_-adrenoceptor antagonist for benign prostatic hyperplasia. Pars plana or anterior partial vitrectomy was performed in all cases. All eyes showed an extremely deep anterior chamber, a concave iris configuration, and contact between the IOL optic and the iris at the pupillary margin. Pupil capture was detected in 2 cases. The mean (± SD) anterior chamber angle (ACA) was 89.91 ± 10.06°, and the anterior chamber depth (ACD) was 4.42 ± 0.16 mm before LPI. After LPI, the iris immediately became flat with a decreased ACA (51.70 ± 2.59°; *P* = 0.018) and ACD (4.14 ± 0.15 mm; *P* = 0.012). After LPI, the intraocular pressure decreased from 19.75 ± 3.77 mmHg to 15.63 ± 4.30 mmHg (*P* = 0.011), and the spherical equivalent decreased from -0.643 ± 0.385 D to − 0.875 ± 0.505 D (*P* = 0.016).

**Conclusion:**

Concomitant vitrectomy, angle pigmentation, and iridodonesis may be risk factors for RPB after scleral-sutured PC IOL implantation. LPI is effective for relieving the RPB.

## Background

Reverse pupillary block (RPB) has been proposed as the causative mechanism for pigment dispersion syndrome [1–7]. In RPB, the iris functions as a flap valve, allowing aqueous humor to pass from the posterior chamber to the anterior chamber but not in the opposite direction [2]. The aqueous humor trapped within the anterior chamber causes posterior bowing of the peripheral iris. Contrary to the characteristic posterior-to-anterior rush of fluid immediately after laser peripheral iridectomy (LPI) in traditional relative pupillary block, the breakthrough fluid rush in RPB is in the reverse direction, indicating that differential pressures exist between the anterior and posterior chambers [3]. Reverse pupillary block is described in phakic patients rather than pseudophakic patients because of the relatively thicker crystalline lens.

Although classically described in phakic patients, RPB had also been reported in other clinical situations, including intraocular lens (IOL) implantation in the ciliary sulcus [3], ‘in-the-bag’ IOL implantation [8, 9], and scleral-sutured posterior chamber (PC) IOL implantation [10, 11]. Anterior segment optical coherence tomography, recently developed and widely used for diagnostic purposes, and Scheimpflug imaging have been helpful in detecting and understanding the mechanism of RPB in a number of clinical cases [9–11].

Reverse pupillary block is rare in scleral-sutured PC IOL, but extreme posterior bowing of the iris can lead to repetitive pupil capture, pigment dispersion due to increased iris-optic contact, and increased intraocular pressure (IOP). In 2009, Higashide et al [10] reported 4 cases of RPB in 3 patients who had undergone scleral-sutured PC IOL implantation; however, the clinical features and factors that contribute to the development of RPB in this situation are not yet fully understood.

Here we report our experience with 8 cases of RPB in 8 patients who had undergone scleral-sutured PC IOL and assess the efficacy of LPI.

## Methods

Between February 2015 and May 2016, 8 pseudophakic patients who had undergone scleral-sutured PC IOL implantation due to subluxation of the crystalline lens or the IOL, which had already been implanted at the local medical center before the visit to our clinic and were found to have RPB after scleral-sutured PC IOL implantation, were included in this retrospective study. Patients with a history of glaucoma and those with a history of vitrectomy, implantation of a scleral-sutured sulcus IOL, one-haptic fixation of a PC IOL, or fixation of an IOL-capsular bag complex were excluded. Data on pre-existing medical conditions, current medication, ophthalmologic surgical history, and history of trauma were collected. All patients underwent an ophthalmic examination, including uncorrected visual acuity, best corrected visual acuity (BCVA, logMAR system), IOP, spherical equivalent (SE), and slit lamp examination. The study was approved by the Institutional Review Board (IRB) of Keimyung University Dongsan Medical Center (IRB no. 2016-05-069) and was performed in accordance with the tenets of the Declaration of Helsinki.

Six eyes had undergone a limited anterior partial vitrectomy through a limbal incision site in conjunction with scleral-sutured PC IOL implantation. Pars plana vitrectomy was performed in 2 eyes and concomitant pars plana lensectomy was performed in one case of crystalline lens subluxation. During anterior partial vitrectomy, a 23-gauge infusion cannula and the cutting tip of the 23-gauge vitrectomy instrument were inserted through two limbal incision sites, followed by removal of the anterior vitreous and vitreous near the IOL. Pars plana vitrectomy was performed after insertion of 3 trocars at the superonasal, superotemporal, and inferotemporal quadrants using a 23-gauge 45-degree stiletto blade (0.72 mm in diameter). The IOLs were repositioned in 5, exchanged in 3, and implanted in 1 of the 8 eyes. PC IOL implantation was performed using an ab externo method in all cases. To minimize IOL decentration during transscleral fixation, we used the toric axis marker and marked the fixation axis. After scleral flaps were prepared in 2 positions 180° apart, a straight needle attached to a 10–0 polypropylene suture for IOL fixation was passed through the bed of half-thickness scleral flaps 2.0 mm posterior to the limbus in a direction parallel to the iris.

Evaluations using OCT/SLO® (OTI, Ophthalmic Technologies Co., Toronto, ON, Canada) with an affordable add-on (AC Cornea) lens were performed to assess quantitative parameters in the anterior segment with respect to RPB. The anterior chamber angle (ACA) was measured in horizontal scans of tomographic images by placing the apex of the ACA tool in the angle recess with its arms parallel to the iris surface and corneal endothelium. The ACA was calculated as the mean of the measurements at the nasal angle and temporal angle (0° and 180°, respectively) and performed using ImageJ (http://imagej.nih.gov/ij/; provided in the public domain by the National Institutes of Health, Bethesda, MD, USA) [12]. Central anterior chamber depth (ACD) and axial length (AL) were measured using A-scan III (Mentor®, Mentor O & O, Inc., Norwell, MA, USA).

The statistical analysis was performed using SPSS version 22.0 software (IBM Corp., Armonk, NY, USA). To compare the corrected distance visual acuity (CDVA), IOP, ACA, ACD, and SE before and after iridotomy, the Wilcoxon signed-rank test was used after a skewness check using the Shapiro-Wilk test because of violation of the normal distribution assumption. The data are reported as the mean G standard deviation; a *p*-value ≤0.05 was considered to be statistically significant.

## Results

**Fig. 1:**
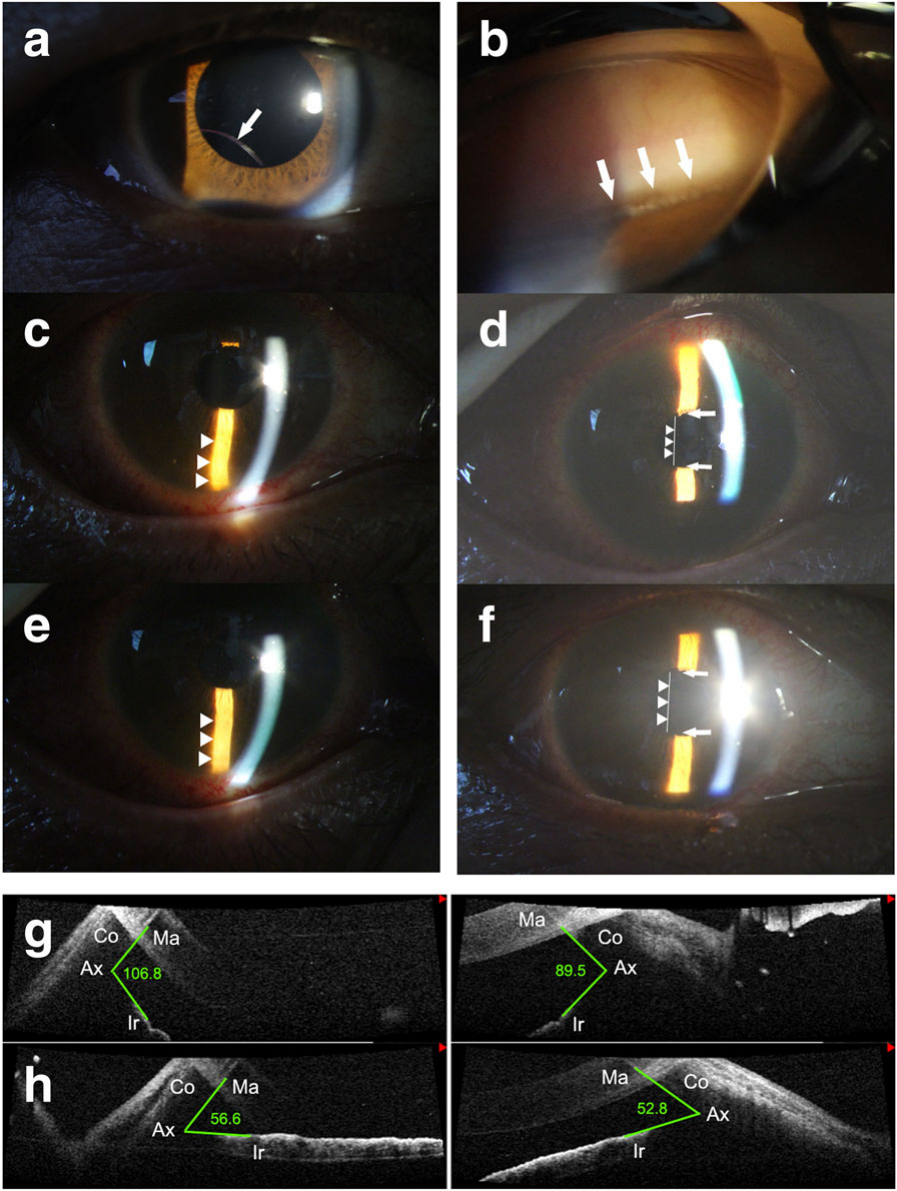
Representative slit lamp photographs and tomographic images (case 8). **a** Preoperative photograph shows inferotemporal subluxation of the intraocular lens (*arrow*). **b** Preoperative gonioscopic image of the iridocorneal angle shows a distinctive pigmentation in the trabecular meshwork (*arrow*). **c** Slit lamp photograph before iridotomy shows a concave iris (*arrowheads*). **d** Slit lamp photograph before iridotomy shows the apposition between the iris and intraocular lens optic (*arrows*), indicating the presence of reverse pupillary block. **e** Slit lamp photograph taken immediately after iridotomy shows a flat iris (*arrowheads*). **f** Slit lamp photograph taken immediately after iridotomy shows the space between the iris and intraocular lens optic (*arrows*), indicating recovery from reverse pupillary block. **g** Tomographic image taken before iridotomy show a concave iris configuration and wide anterior chamber angle. **h** Tomographic image taken immediately after iridotomy shows a flat iris and a narrower anterior chamber angle. The arrowheads and *white* line in **d** and **f** indicate the posterior surface of the intraocular lens. (Ax = angle, Co = cornea, Ir = iris, Ma = mirror artifact)

Table 1 summarizes the demographic and clinical characteristics of the study participants. Seven men and one woman who underwent LPI a mean of 6.25 ± 6.94 months (range 5 days to 21 months) after scleral-sutured PC IOL implantation were included. The mean AL was 24.38 ± 0.66 (range 23.55–25.63) mm. Angle pigmentation was observed in all cases by gonioscopy, and manifested as mild pigmentation in 5 eyes and moderate pigmentation in 3 eyes. Four eyes had iridodonesis and 2 of the 7 men had taken a systemic α_1A_-adrenoceptor antagonist (tamsulosin) for benign prostatic hyperplasia. In all cases, a three-piece IOL had been used, supported by a polymethyl methacrylate haptic design. Acrylic was used as the optic material in 5 cases and silicone in 3 cases. All the eyes had an extremely concave iris configuration, a deep anterior chamber, and pupil-IOL contact. In all cases, RPB was treated immediately after identifying the existence of RPBs using neodymium: YAG LPI in the peripheral iris at 11 o’clock in the right eye or 1 o’clock of the left eye, and a distinctive backward flow of pigment was observed when perforation of the iris was complete. After LPI, resolution of RPB was confirmed in all cases when the iris became flat and the distance between the iris and the IOL became deeper (Fig. 1).

**Table 1:** Patient demographics and clinical characteristics

Patient	Gender	Surgery-LPI^e^ (months)	AL (mm)	Surgery^f^	Pupil capture	Angle pigment	Iridodonesis	Tamsulosin	Intraocular lens details (Diopters, model name, optic material, edge design)
1	M	6	23.71	PPV + PPL + IOL implant (P)	−	+	+	+	+19.5, PC − 60AD^a^, acrylic, square
2	M	21	24.12	IOL reposition + Ant vitrectomy (S)	−	+	−	+	+20.5, LI61U^b^, silicone, round
3	M	3	24.44	IOL exchange + Ant vitrectomy + IOL reposition (#2) (S)	+	+	−	−	+17.5, PC − 60AD^a^, acrylic, square
4	F	12	24.86	IOL reposition + Ant vitrectomy (S)	+	+	+	−	+16.0, VA60BBR^a^, acrylic, square
5	M	3	23.55	IOL reposition + Ant vitrectomy (S)	−	+	+	−	+20.5, SI − 30NB^c^, silicone, round
6	M	1	24.38	IOL exchange + Ant vitrectomy (S)	−	+	−	−	+18.0, Sensar® AR40e^d^, acrylic, square
7	M	3	24.33	IOL exchange + Ant vitrectomy (S)	−	+	+	−	+17.0, Sensar® AR40e^d^, acrylic, square
8	M	1	25.63	PPV + IOL reposition (S)	−	+	−	−	+14.0, LI61U^b^, silicone, round

^a^Manufactured by HOYA Corporation, Tokyo, Japan; ^b^Bausch & Lomb, Rochester, NY, USA; ^c^Allergan Inc, Irvine, CA, USA; ^d^Advanced Medical Optics, Santa Barbara, CA, USA. ^e^surgery – LPI = time interval between surgery and LPI. ^f^P = primary surgery; S = secondary surgery. Abbreviations: *AL* axial length, *Ant* anterior, *F* female, *L* left eye, *LPI* laser peripheral iridotomy, *M* male, *R* right eye, *PPL* pars plana lensectomy, *PPV* pars plana vitrectomy

Table 2 compares the CDVA, IOP, ACA, ACD and SE before and after settling of the RPB. Mean CDVA improved from 0.25 ± 0.37 to 0.19 ± 0.38 (*P* = 0.059) and mean IOP decreased significantly from 19.75 ± 3.77 (range 14–26) mmHg to 15.63 ± 4.30 (range 10–20) mmHg (*P* = 0.011). Mean ACA and ACD also decreased significantly from 89.91 ± 10.06 (range 70.5–100.7) degrees to 51.70 ± 2.59 (range 48.1–54.7) degrees, and from 4.42 ± 0.16 (range 4.18–4.72) mm to 4.14 ± 0.15 (range 3.86–4.29) mm (*P* = 0.018 and *P* = 0.012, respectively). Mean SE decreased significantly from − 0.643 ± 0.385 (range − 1.25,−0.25) D to − 0.875 ± 0.505 (range − 1.75,−0.375) D, demonstrating anterior IOL shift (*P* = 0.016).

**Table 2:** Changes in parameters caused by reverse pupillary block

No	CDVA		IOP (mmHg)		ACA (degrees)		ACD (mm)		SE (diopters)		
	RPB+	RPB–	RPB+	RPB–	RPB+	RPB–	RPB+	RPB–	RPB+	RPB–	Change
1	0	0	22	19	86.3	48.6	4.44	4.29	−0.375	−0.50	−0.125
2	0	0	21	20	91.9	51.7	4.28	4.08	−0.25	−0.375	−0.125
3	1.1	1.1	17	14	−^a^	−^a^	4.33	4.17	−^b^	−^b^	−^b^
4	0	0	14	12	87.3	53.2	4.45	4.28	−0.5	−0.625	−0.125
5	0.2	0.1	22	19	70.5	54.2	4.31	4.10	−0.5	−0.625	−0.125
6	0.2	0.1	26	20	100.7	48.1	4.18	3.86	−1.125	−1.375	−0.25
7	0.1	0	19	10	94.5	51.4	4.66	4.21	−0.50	−0.875	−0.375
8	0.4	0.2	17	11	98.2	54.7	4.72	4.12	−1.25	−1.75	−0.5
Mean	0.25	0.19	19.75	15.63	89.91	51.70	4.42	4.14	−0.643	−0.875	−0.232
*P*-value^‡^	0.059		0.011		0.018		0.012		0.018		

^a^No measurement of anterior chamber angles at Scheimpflug images; ^b^uncheckable due to corneal state (slightly edematous cornea); ^‡^Wilcoxon signed rank test. Abbreviations: *CDVA* corrected distal visual acuity, *LPI* laser peripheral iridotomy, *IOP* intraocular pressure, *ACA* anterior chamber angle, *ACD* anterior chamber depth, *SE* spherical equivalent, *RPB*+ in the presence of reverse pupillary block, *RPB*– in the absence of reverse pupillary block, *R* right eye, *L* left eye

## Discussion

RPB is a rare postoperative complication of scleral-sutured PC IOL implantation reported by some authors [10, 11]. In addition, Khng et al [13] reported 2 eyes with intermittent pupil capture as a result of RPB. Pupil capture is an early complication of scleral-sutured PC IOL implantation, and possibly a severe or advanced form of RPB. In some of the published reports concerning pupil capture, vitrectomy was performed with scleral-sutured PC IOL implantation [10, 13–15]. The proposed mechanism in cases with a well-positioned scleral-sutured PC IOL is posterior bowing of the iris that pushes the IOL until pupil capture occurs [10, 13]. Bading et al [15] reported pupil capture in 6 eyes (9.6%) after combined pars plana vitrectomy and scleral-sutured PC IOL implantation, and Johnston et al [14] found that intermittent pupil capture was the most common complication (9 eyes, 14.3%) in the early postoperative period. This complication is usually transient; it can be treated with pupil dilation and its recurrence might be prevented using miotic agents. However, in the study by Johnston et al. [14], 2 cases required surgical repositioning of the IOL optic. Khng et al [13] reported 2 eyes with previous vitrectomy and well-positioned PC IOL that developed intermittent pupil capture and recommended performing an Nd: YAG LPI to prevent or reduce the risk of recapture when a miotic agent is not favored or is poorly tolerated.

**Fig. 2:**
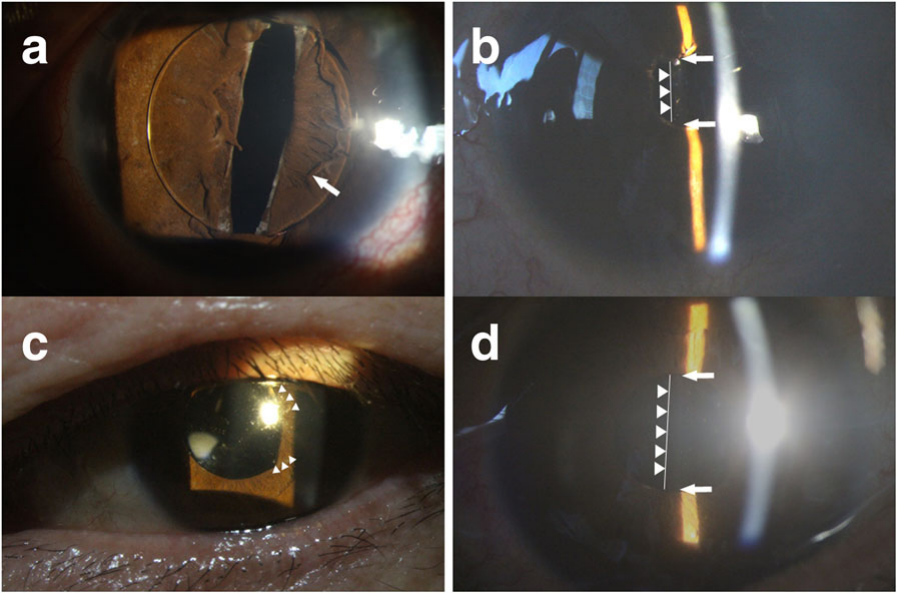
Slit lamp photographs in cases 3 (**a**, **b**) and 4 (**c**, **d**). **a** Slit lamp photograph taken 1 month postoperatively shows dislocation of the intraocular lens anterior to the iris (*arrow*). **b** Slit lamp photograph taken after iridotomy shows the space between the iris and intraocular lens optic (*arrows*). **c** Slit lamp photograph taken 1 month postoperatively shows partial pupil capture (*arrowheads*). **d** Slit lamp photograph taken after iridotomy shows the space between the iris and intraocular lens optic (*arrows*). The arrowheads and *white line* in **b** and **d** indicate the posterior surface of the intraocular lens

In our study, 2 cases of pupil capture developed and LPI was performed in these 2 eyes (Fig. 2). In case 3, we performed an IOL exchange due to malposition of a scleral-sutured PC IOL, but this patient developed dislocation of the IOL anterior to the iris on two occasions; despite repositioning of the IOL and use of pilocarpine 2% eyedrops four times per day, recurrent anterior dislocation of IOL developed, but no recurrence of anterior dislocation or pupil capture was observed after LPI. In case 4, partial pupil capture occurred twice during follow-up. Despite the use of pilocarpine 2% 4 times daily, recurrent partial pupil capture developed, but no recurrence of pupil capture was observed after LPI.

In this study, tomographic images confirmed the extremely concave iris configuration associated with RPB and allowed quantitative evaluation of the change in iris concavity, assessed by ACA measurements, before and after resolution of RPB. We speculate that the cornea could be also concave because of entrapment of aqueous humor in the anterior chamber as a result of impaired aqueous outflow before LPI and become flatter after LPI. However, the concavity of the cornea showed no significant change, which may be due to the rigidity of the cornea itself or due to the distortion of the cornea by the add-on contact lens of the OCT used for analyzing the anterior segment. The mean change in ACA was 38.21° in 7 eyes, which is slightly larger than the 33.18° reported by Higashide et al [10]. Further, the mean ACD change in the 8 eyes was 0.28 mm, which is less than the 0.47 mm in the study by Higashide et al [10], who reported that the amount of posterior movement of IOL optics varied significantly between cases, and this may be related to the design of and materials used in the IOLs. As in our study, Higashide et al [10] performed scleral-sutured PC IOL implantation 2.0 mm posterior to the limbus (the bag position) and used three-piece IOLs in all their 4 cases. This discrepancy in ACD change between the two studies may again be due to differences in IOL design. In our study, silicone IOLs were used in cases 2, 5, and 8 and acrylic IOLs in cases 1, 3, and 4, with 5° of haptic angulation for all IOLs. Cases 2, 5, and 8 had greater deepening of the anterior chamber than the other cases, possibly because the relatively less rigid silicone IOL may be more vulnerable than the acrylic IOL to being pushed posteriorly by the RPB. As well as the IOL design, a change in IOP could also be associated with a change in ACD. Despite having acrylic IOLs, cases 6 and 7 showed a greater change in ACD than the other cases, suggesting that a larger decrease in IOP may be involved.

The mean change in SE was 0.232 D in 7 eyes after resolution of RPB, which represented a statistically significant change and was much less than the 0.68 D reported by Higashide et al [10]. This indicates a less anterior shift of the IOL and may be related to surgeon preferences with regard to IOL design, surgical method used, and the suture tension of scleral-sutured PC IOL. Axial movement of an IOL can cause visual symptoms resulting from a refractive change. Using a data set of 7418 eyes, Olsen [16] found that a refractive change caused by a change in the ACD is greater in eyes with a shorter AL based on the relationship between AL and the IOL prediction error resulting from a 0.25 mm error in postoperative ACD. According to Olsen [16] a 0.25 mm change in ACD will generally cause a refractive change of approximately 0.30 D in eyes with an AL of 24.0 mm (similar to our cases) and 0.10 D in eyes with an AL of 30.0 mm. Although RPB led to relatively small hyperopic shifts in our cases, such shifts can be considerable in eyes with a shorter AL. For example, according to Olsen [16] a 1.0 mm deepening of the anterior chamber in an eye with a short AL of 21.0 mm will cause a posterior shift of 2.00 D. This shift may be symptomatic, so must be treated.

Several risk factors for RPB after scleral-sutured PC IOL implantation have been reported, one of which is a flaccid iris [9, 11, 17, 18]. A flaccid iris acts as a check valve, preventing movement of the aqueous humor from the anterior to posterior chamber and results in reverse pressure gradients across the anterior and posterior chamber [18]. Marked iridodonesis, which was present in 4 cases in our study and in one case in the study reported by Rhéaume et al [8], might suggest a flaccid iris. Flaccid iris is one of the triad of signs found in the intraoperative floppy iris syndrome (IFIS) [19] and a strong association between occurrence of IFIS and use of tamsulosin, a systemic α_1A_-adrenoceptor antagonist, has also been reported [19, 20]. Although signs of IFIS were not obvious during fixation surgery in our patients, it is possible that flaccid iris was latent in 2 patients who received tamsulosin in our study. In case 3, IOL subluxation developed with extensive zonular dialysis in the right eye, despite the patient being a young man with no history of trauma. This patient was right-handed and had a habit of frequent eye rubbing. Previous studies have reported that habitual eye rubbing can induce zonular rupture and IOL subluxation or dislocation [21–23]. Agrawal et al [24] reported a case of iridoschisis associated with lens subluxation and postulated that the lens subluxation precipitated iridoschisis by mechanical rubbing of the back of the iris, which may be associated with flaccid iris.

Some authors have argued that in the vitrectomized eye, in conjunction with a flaccid iris, RPB after scleral-sutured PC IOL implantation is caused by increased flow of aqueous humor from the posterior chamber and the vitreous cavity to the anterior chamber with movement of the eye due to the absence of the lens capsule, the lens zonular fibers, and the vitreous [9, 11]. In our study, all cases had partial vitrectomy or pars plana vitrectomy and may have developed RPB via this mechanism. Further, some studies have reported that high axial myopia might also be a cause of RPB [9, 11, 14]. A highly myopic eye tends to have a greater posterior chamber volume, leading to more aqueous humor flowing into the anterior chamber and a flaccid iris, but this has not yet been established [9]. All cases in our study had a relatively long axial length.

In our study, all cases showed angle pigmentation at preoperative gonioscopy. Interestingly, all cases in our study had severe preoperative zonular dialyses; consequently we should have performed scleral sutured PC IOL implantation instead of the in-the-bag placement, even with the support of a capsular tension ring. Mechanical factors such as crystalline lens or IOL tilt and variable axial position due to zonular weakness might induce contact between the crystalline lens or IOL optic and the middle posterior iris pigment epithelium, leading to release of pigment. This accumulated pigment might contribute to impaired aqueous flow through the trabecular meshwork, and resistance to aqueous outflow might be a risk factor of RPB. A squared-edge IOL design has been reported to be a risk factor for chafing of the iris in the absence of the lens capsule [25–27] but preoperative angle pigmentation was not aggravated by IOLs with a squared-edge design after scleral-sutured PC IOL implantation in our study.

Considering all potential risk factors together, in circumstances of impaired aqueous outflow due to angle pigmentation, increased flow of aqueous humor from the posterior chamber and the vitreous cavity to the anterior chamber might cause entrapment of aqueous humor in the anterior chamber, followed by RPB due to back bowing of a flaccid iris after scleral-sutured PC IOL implantation, especially in an eye that is vitrectomized and/or has a long axial length.

LPI has been reported to be effective in relieving RPB after scleral-sutured PC IOL implantation [8–11]. All of the patients in our study responded immediately to LPI, with significant improvements in vision, IOP, and other biometrics. Contrary to the characteristic posterior-to-anterior rush of fluid immediately after LPI in traditional relative pupillary block, the breakthrough fluid rush in RPB is in the reverse direction. Physiologically, with this stasis of aqueous humor in the anterior chamber, whereby differential pressures exist between the anterior and posterior chambers, creation of an iridotomy allows rapid resolution of RPB, as the pressures across the anterior and posterior segments then freely equilibrate.[4] This restores the planar configuration of the iris, relieving posterior bowing and RPB [4, 28–30]. Our post-LPI tomographic and Scheimpflug images revealed successful flattening of the iris and widening of the space between the posterior iris and IOL. This was noted to be secondary to the forward shift of the iris and the stable IOL position in the posterior chamber after treatment. In addition, all measurements in our study, including CDVA, IOP, ACA, ACD, and SE, showed a statistically significant improvement after LPI and demonstrated the efficacy of LPI.

The main limitation of this study is that we did not carry out a comparison with a control group of eyes that underwent scleral-sutured PC IOL implantation but did not develop RPB. RPB has been considered a rare postoperative complication after scleral-sutured PC IOL implantation. Further investigations, including a case–control study, may be necessary to calculate a risk ratio based on the risk factors and address the incidence of RPB after scleral-sutured PC IOL implantation. However, most of the increased IOP or pupil capture might occur via the above-mentioned mechanisms and RPB might be an underestimated phenomenon rather than a rare one. Additionally, there were no specific presenting symptoms in our cases, except 2 pupil capture cases that presented with blurred vision only; the RPBs were diagnosed only on regular follow-up. Routine postoperative examination of IOP and ACD using anterior segment optical coherence tomography or Scheimpflug imaging can be considered. In addition, in patients with a high preoperative IOP and severe angle pigmentation on gonioscopy, preoperative or intraoperative evidence of a flaccid iris and a long AL, a careful, meticulous anterior vitrectomy, if necessary, should be performed during surgery, given the risk for RPB. In these patients, intraoperative prophylactic peripheral iridectomies using a vitreous cutter or postoperative LPI may prevent the complications induced by RPB, such as increased IOP, pupil capture, positional instability of the IOL, and refractive change.

## Conclusion

Concomitant vitrectomy, angle pigmentation, and iridodonesis may increase the risk for RPB after scleral-sutured PC IOL implantation, and LPI is effective in relieving the RPB.

## Abbreviations

ACA: Anterior chamber angle
ACD: Anterior chamber depth
AL: Axial length
BCVA: Best corrected visual acuity
CDVA: Corrected distance visual acuity
IFIS: Intraoperative floppy iris syndrome
IOL: Intraocular lens
IOP: Intraocular pressure
LPI: Laser peripheral iridectomy
PC: Posterior chamber
RPB: Reverse pupillary block
SE: Spherical equivalent
